# A Manual of Chemistry

**Published:** 1846-03

**Authors:** 


Art. IX.—*A Manual of Chemistry.* By Richard D. Hoblyn, A. M. Oxon. Author of a Dictionary of Terms used in Medicine and the Collateral Sciences. New York: Samuel S. & William Wood. 1846. (18mo. pp. 335.)

In this little volume, Chemistry is exhibited in the utmost state of condensation, its leading facts and principles being brought within the compass of a pocket-volume. Concise as is Fownes's "Chemistry for Students," the work of Hoblyn is shorter. And yet it is lucid, and satisfactory, as far as it goes, forming a manual which the young student will consult with great advantage. Having mastered some such elementary treatise as this, the student is prepared to take up the more elaborate systems of chemistry; but our observation is that most minds are overpowered by the large works when put to their study without any preparatory training, and that, in this way, a distaste not unfrequently is excited to the science. If the learner be very young, let him enter upon the study with Hoblyn for his guide; if more advanced in years and learning, let him begin with the elements of Fownes, and subsequently study Kane, Graham or Turner; and thus, conducted by easy and natural steps, he will be captivated by the developments of a science so rich in wonders, and so manifestly practical and useful, instead, as is now too often the case, of throwing aside his books in despair of ever comprehending its truths. The manual before us is well adapted to the wants of those who are to prosecute the study without the aid of an instructor. It is illustrated by wood-cuts, and gives directions for manipulation sufficiently full for a work of the sort. In a word, it is well suited to the class of beginners, and doubtless will be extensively introduced into schools.

